# Flame-Made Doped Iron Oxide Nanoparticles as Tracers for Magnetic Particle Imaging

**DOI:** 10.1021/acs.chemmater.5c00331

**Published:** 2025-05-20

**Authors:** Shaquib Rahman Ansari, Eric Daniel Imhoff, Yael del Carmen Suárez-López, Andrii Melnyk, Carlos M. Rinaldi-Ramos, Alexandra Teleki

**Affiliations:** † Department of Pharmacy, Science for Life Laboratory, 8097 Uppsala University, 75123 Uppsala, Sweden; ‡ Department of Chemical Engineering and J. Crayton Pruitt Family Department of Biomedical Engineering, 3463 University of Florida, Gainesville, Florida 32611-6005, United States

## Abstract

Magnetic particle imaging (MPI) is an emerging imaging modality that shows potential in tumor imaging, cell tracking, and angiography. It uses the signal generated from superparamagnetic iron oxide nanoparticles (SPIONs) with zero attenuation in tissue, showing excellent sensitivity and contrast. MPI resolution and sensitivity are dependent on the nonlinear dynamic magnetization of the SPION tracer and can be improved by tuning their magnetic properties. Doping SPIONs with manganese or zinc is an effective and biocompatible route to modify the magnetic properties of SPIONs. This study developed SPIONs doped with manganese or zinc as MPI tracers using flame spray pyrolysis (FSP), a highly scalable synthesis technique. The MPI performance was evaluated with a MOMENTUM imager. Postsynthesis citrate coating and filtration significantly enhanced the MPI resolution of SPIONs. The Zn-doped SPIONs exhibited the best resolution, while Mn-doped SPIONs showed the highest sensitivity. The overall MPI performance of all tracers was closely linked to their magnetic diameter and susceptibility, but deviated noticeably from the predictions of the Langevin model. Zn-doped SPIONs were encapsulated in a water-dispersible nanocarrier using flash nanoprecipitation (FNP), circumventing the need for citrate coating while preserving MPI performance. These findings show that the hydrodynamic size, size distribution, and composition of the SPIONs are critical to MPI performance and highlight the potential of combining FSP and FNP for large-scale production of the MPI tracers.

## Graphical abstract

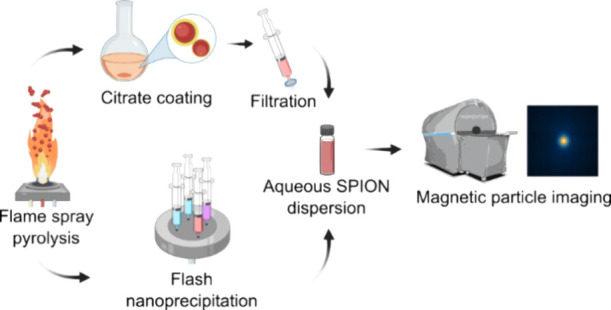

Magnetic particle imaging (MPI) is a promising imaging modality with potential medical applications in imaging of inflamed vasculature, blood perfusion, solid tumors, and cell-based therapeutics.^1,2^ MPI uses the nonlinear magnetic response of superparamagnetic iron oxide nanoparticles (SPIONs) to an external magnetic field with no signal contribution from surrounding tissue. This technique provides exceptional sensitivity, with a signal that is directly proportional to the iron mass, enabling precise quantification of the tracer.^3^ In MPI, a strong magnetic field gradient is applied in the field of view to create a small so-called field-free region, outside of which particles are saturated. An oscillating uniform magnetic field is superimposed, generating a magnetization response solely from the tracers in the field-free region. This response can be measured using induction coils and processed by two MPI image reconstruction techniques: x-space or harmonic-space.

In x-space MPI, as implemented in the commercial MOMENTUM MPI scanner (Magnetic Insight, Inc., USA), the signal is modeled as the convolution of the nanoparticle spatial distribution with the point spread function (PSF) of the tracer. The sensitivity and resolution of MPI tracers can be estimated by the intensity (*I*) and full-width at half-maximum (fwhm) of the PSF, respectively. Tracer performance is influenced by extrinsic factors such as the strength of magnetic field gradient, amplitude and frequency of the oscillating magnetic field, and tracer properties such as saturation magnetization (*M*_s_), coercivity (*H*_c_), particle size, and anisotropy.^4^ While MPI demonstrates high sensitivity, improvements in tracer resolution could enable novel applications and facilitate clinical translation.^5^ Additionally, more sensitive tracers with enhanced resolution can lower the magnetic field gradient strength required to achieve a target resolution, thus reducing the costs for clinical implementation.

Development of new MPI tracers is often guided by the Langevin model, which does not fully account for tracer properties such as polydispersity, anisotropy, relaxation time, crystallinity, and presence of multidomain structures.^6^ Moreover, increasing the particle size also increases the relaxation effects, which in turn degrade the MPI performance.^7^ Efforts to enhance the performance of MPI tracers have focused on optimizing nanoparticle size, minimizing the magnetically dead layer, improving crystallinity and phase purity, and ensuring stable colloidal dispersion.^1^ In addition, recent studies have shown improved MPI performance by doping SPIONs with cobalt, nickel, zinc, or manganese.^8–11^ Previous investigations have demonstrated that the substitution of Fe^2+^ ions of the magnetite lattice with Zn^2+^ or Mn^2+^ can influence magnetic properties.^12–15^ Relaxometry measurements revealed that Mn and Zn-doped SPIONs reduced relaxation time and fwhm, potentially improving resolution in MPI applications.^11,16,17^ However, successful clinical implementation of doped ferrites requires scalable manufacturing techniques that ensure precise control over SPION composition and batch-to-batch reproducibility, while maintaining the sensitivity, resolution, and biocompatibility critical for MPI.^18^

Conventional synthesis methods, such as coprecipitation and thermal decomposition, are effective in producing SPIONs with complex stoichiometry at laboratory scale, but face challenges in scaling up.^1^ In contrast, flame spray pyrolysis (FSP) offers a scalable and reproducible approach for the synthesis of doped SPIONs,^19–21^ capable of producing up to 12.5 kg h^–1^ of nanoparticles at pilot scale.^22^ In our previous study, we applied quality by design principles to FSP and presented its ability to synthesize high-performance doped SPIONs for magnetic hyperthermia.^19^ We also showed that flame-made SPIONs can be readily coated with citrate to yield stable aqueous suspensions suitable for biological applications. The well-documented ability of FSP to control size and composition makes it well-suited to systematically study how physicochemical and magnetic properties of nanoparticles influence MPI performance.^21,23–25^

In this work, we systematically investigated the effect of the dopant composition and nanoparticle size on the MPI performance of SPIONs. The SPIONs were produced by FSP and doped with Mn and Zn at varying concentrations and nanoparticle sizes (Figure 1). The synthesized nanoparticles were coated with citrate and filtered to obtain stable aqueous suspensions. We evaluated the structural, colloidal, and magnetic properties of the nanoparticles both in their solid state and in aqueous suspension. The cytotoxicity of doped SPIONs was investigated in mammalian cells. As a scalable and facile alternative to citrate coating, we also demonstrated the use of flash nanoprecipitation (FNP) with a multi-inlet vortex mixer (MIVM) to manufacture aqueous SPION dispersions. The MPI intensity and resolution of all tracers were evaluated with 2D scans using a MOMENTUM MPI scanner and compared to the commercially available tracers ferucarbotran and VivoTrax+. Overall, this study reports a systematic investigation of the effect of the nanoparticle size and composition on its MPI performance. The combination of FSP with FNP represents a scalable approach for producing aqueous suspensions of doped ferrites optimized for MPI.

**Figure 1:**
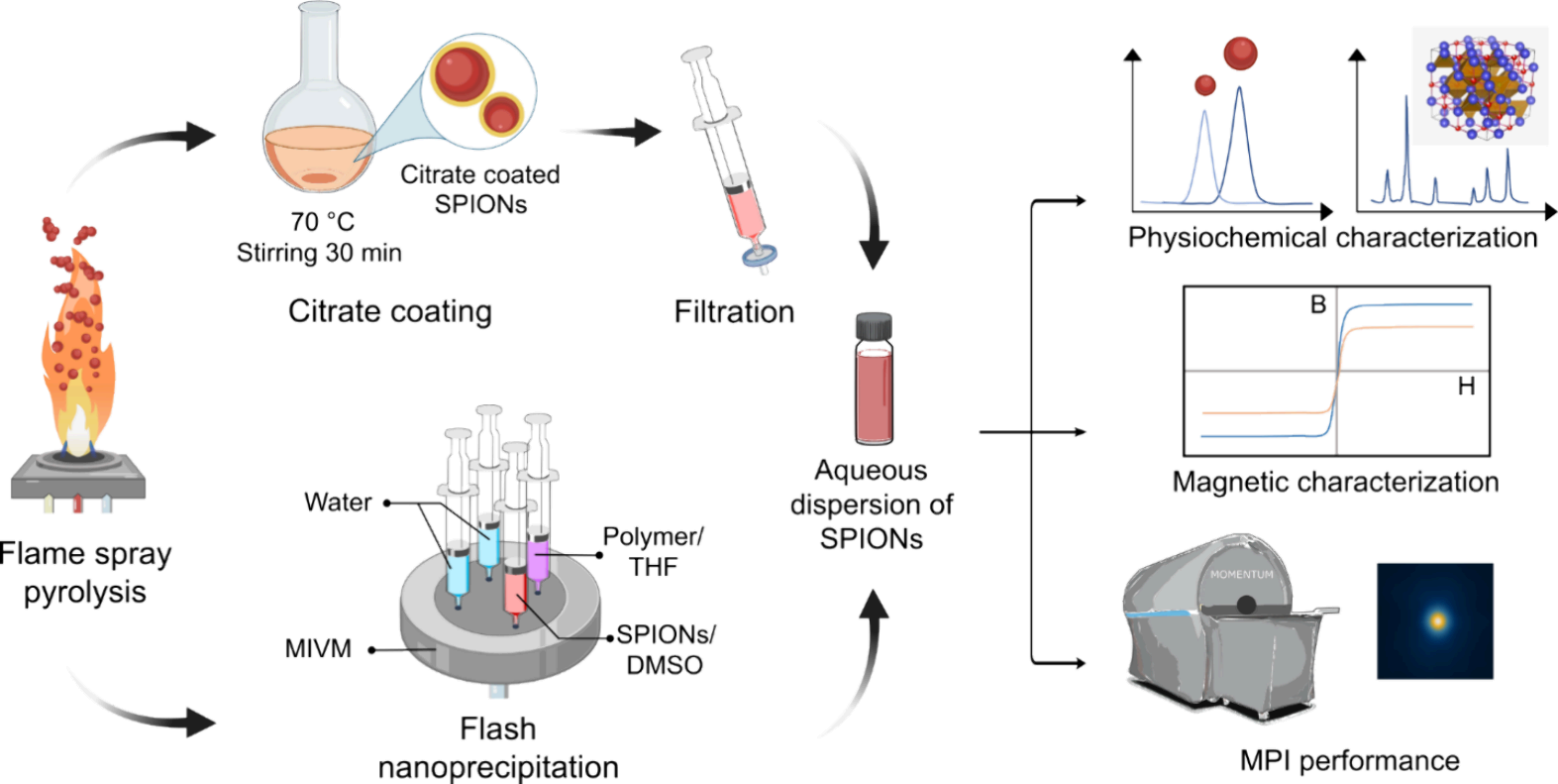
Schematic illustration of the development of flame-made tracers for magnetic particle imaging (MPI). The superparamagnetic iron oxide nanoparticles (SPIONs) were synthesized by flame spray pyrolysis (FSP). Aqueous dispersions of SPIONs were produced by citrate coating and subsequent filtration. Rapid production of aqueous SPION suspensions was achieved by flash nanoprecipitation (FNP) using a multi-inlet vortex mixer (MIVM). FNP enabled mixing of antisolvent (water) with the SPION dispersion in dimethyl sulfoxide (DMSO) and the polymer solution in tetrahydrofuran (THF). The aqueous SPION suspensions were characterized for their physicochemical properties, magnetic behavior, and performance as MPI tracers.

## Results and Discussion

### Synthesis and Characterization of SPION Powders

The impact of SPION composition on MPI performance was investigated by using FSP-made nanoparticles doped with varying concentrations of Zn or Mn (Table S1). Computational and experimental studies have shown that MPI tracers reach an optimal resolution at a critical diameter of 20–30 nm, beyond which relaxation effects worsen resolution.^26,27^ Additionally, optimal MPI resolution is reported to occur for the largest SPION nanocrystals with a diameter below the ferromagnetic limit.^28^ Therefore, to investigate this with FSP-made SPIONs, the crystal size (*d*_XRD_) was controlled through FSP parameters, yielding particles from 13–17 nm (small) to 21–33 nm (large) (Table S2). The small tracers were expected to be well within the superparamagnetic regime, whereas the large nanoparticles were expected to lie at the boundary of the superparamagnetic limit. The phase composition and crystal size of the nanoparticles were analyzed by X-ray diffraction (XRD). All ferrites exhibited diffraction peaks corresponding to cubic spinel structures. The six prominent peaks from the (220), (311), (400), (422), (511), and (440) crystallographic planes (Figure S1a,b), indicate that the particles exhibit the maghemite or magnetite phase. XRD cannot reliably distinguish between the maghemite and magnetite phases due to their overlapping peak positions and similar intensities. However, Mössbauer spectroscopy from our previous study showed that flame-made undoped SPIONs exhibit maghemite phase, while doped ferrite nanoparticles likely show magnetite-like phase.^19^ No peaks indicative of other iron oxide phases (such as wüstite and hematite) or metal oxides of zinc and manganese were detected in any of the SPIONs. Introduction of dopants induced a slight shift in the XRD pattern of SPIONs, suggesting dopant incorporation into the iron oxide crystal lattice.^29,30^ The average crystal size (*d*_XRD_) derived from the XRD pattern aligned with the expected size range of the small and large ferrite nanoparticles, and closely matched their grain size (*d*_BET_) derived from the specific surface area (Table S2).

### Characterization of Aqueous Suspensions of Nanoparticles

The spatial resolution of MPI tracers depends on the uniformity of their size distribution.^2^ Polydispersity is undesirable due to the size dependence of signal strength and resolution. Additionally, agglomeration of nanoparticles can lead to interparticle interactions and magnetic coupling, greatly affecting the magnetic properties of SPIONs and consequently their MPI performance.^31,32^ In a previous study, we demonstrated improved dispersibility of FSP-made SPIONs by citrate coating.^19^ Therefore, to reduce aggregation and enhance the stability of MPI tracers, the SPION surfaces were coated with citrate, followed by filtration to remove residual aggregates, resulting in a narrower size distribution. Overall, this might minimize the Brownian relaxation effects associated with large hydrodynamic diameter particles, allowing improved MPI performance.^28^ Additionally, sodium citrate is categorized as generally recognized as safe (GRAS) by the US Food and Drug Association, warranting its use for biomedical applications.^33^

Aqueous suspensions of most uncoated particles exhibited a multimodal size distribution (70–5000 nm) that rapidly sedimented. The coated nanoparticles showed highly negative zeta potential, which could be attributed to physisorption and chemisorption of citrate ions on the SPION surface (Table 1).^34^ Figure 2a illustrates the hydrodynamic size after citrate coating, with and without filtration. The coated nanoparticles with larger crystal sizes showed markedly larger hydrodynamic sizes (100–700 nm) than SPIONs with smaller crystal sizes (70–85 nm). This is likely due to aggregates formed during high-temperature flame synthesis. Such aggregates are highly resistant to separation even with high-energy ultrasonication or steric stabilization techniques.^35^ Filtration greatly reduced the polydispersity of the citrate-coated SPIONs, resulting in monomodal distributions with a polydispersity index (PDI) of 0.08–0.2 for nearly all tracers except for the large Mn and Zn ferrite nanoparticles (Table 1). After filtration, the zeta potential of all tracers became more negative, with the largest changes observed in the large-sized nanoparticles (Table 1). This reduction in zeta potential could possibly be due to the removal of poorly coated aggregates upon filtration, improving the electrostatic stability of suspensions.

**Table 1:** Hydrodynamic Diameter (*d*_H_), Zeta Potential (ζ), and Polydispersity Index (PDI) of the Tracers in Aqueous Suspension

	*d*_H_ [nm]		ζ [mV]		PDI	
SPION composition	coated	coated and filtered	coated	coated and filtered	coated	coated and filtered
γ-Fe_2_O_3_ (S)	85.4	75.4 ± 2.5	–33	–38.7	0.159	0.108
γ-Fe_2_O_3_ (L)	188.4	170.4 ± 15.3	–15	–39	0.338	0.348
Zn_0.5_Fe_2.5_O_4_ (S)	69.2	65.4 ± 1.8	–28.5	–30.2	0.115	0.128
Zn_0.5_Fe_2.5_O_4_ (L)	701.5	112.2 ± 3.4	–16.5	–40.4	0.358	0.205
Mn_0.25_Fe_2.75_O_4_ (S)	85	72.5 ± 0.9	–26	–38.2	0.164	0.109
Mn_0.25_Fe_2.75_O_4_ (L)	243.8	69.8 ± 1.4	–14.9	–37.7	0.256	0.103
Mn_0.5_Fe_2.5_O_4_ (S)	75.8	68.4 ± 2			0.158	0.085
Mn_0.5_Fe_2.5_O_4_ (L)	294	65.4 ± 1.1			0.282	0.086

**Figure 2:**
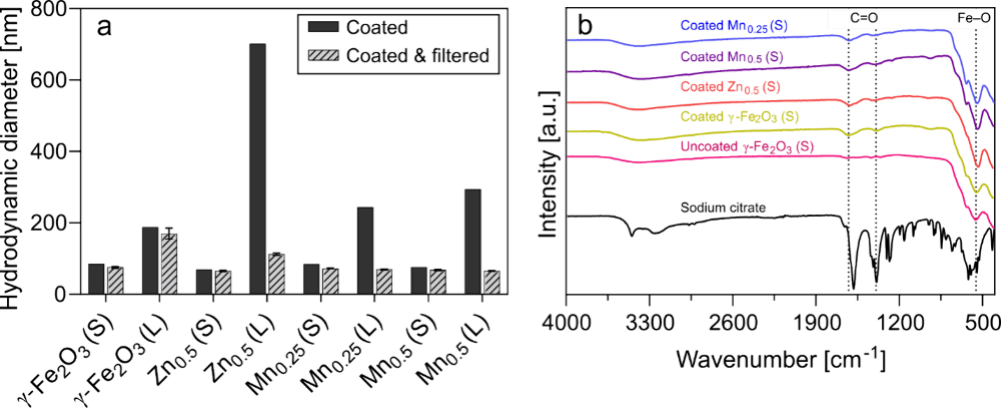
Physicochemical Characterization of aqueous suspensions of SPIONs. (a) Hydrodynamic size of doped citrate-coated nanoparticles before and after filtration. Filtered suspensions were measured in triplicates (±SD). (b) Fourier-transform infrared (FTIR) spectra of small-sized citrate coated SPIONs.

The citrate coating on the surface of small SPIONs was studied by using Fourier-transform infrared (FTIR) spectroscopy (Figure 2b). The citrate-coated nanoparticles showed peaks of Fe–O vibrations at 555 cm^–1^ corresponding to the iron oxide phase.^36,37^ Additionally, peaks at 1625 and 1396 cm^–1^, indicative of the asymmetric and symmetric stretching of carboxyl groups, suggest the presence of citrate coating on SPIONs.^38^ The observed shift in the asymmetric stretching mode toward a lower wavenumber region suggests a mainly hydrogen-bonded system.^39^ Notably, the absence of a band typical for the free carboxylic acid group at around 1700 cm^–1^ (in comparison to the spectrum of tribasic sodium citrate) further indicates complete coordination of all carboxyl groups of the citrate molecule.

### Cytotoxicity of Doped Ferrite Tracers

The choice of MPI tracer should be guided by its potential toxicity, which is influenced by several factors such as size, coating, and core composition.^40^ Previous studies have shown that smaller SPIONs (10 nm) tend to exhibit greater cytotoxicity than their larger (30 nm) counterparts.^40,41^ The relationship between nanoparticle size and toxicity is complex and likely influenced by factors including nanoparticle surface area, cellular uptake mechanisms, and stability in biological media. Therefore, to reduce the confounding effects of size variability and better isolate the impact of core composition on cytotoxicity, we restricted our investigation to small doped ferrites.

Cytotoxicity of the MPI tracers was evaluated in the Madin-Darby canine kidney (MDCK) cell line, a well-established mammalian cell model for toxicity studies in preclinical pharmaceutical development.^42,43^ Given that tracers and contrast agents could potentially result in renal complications, MDCK cells serve as an ideal model system to investigate the biocompatibility of MPI tracers. Figure 3 shows the dose-dependent viability of MDCK cells following 24 h of exposure to the small-sized undoped and doped SPIONs. At a concentration of 100 μg mL^–1^, Zn-doped SPIONs were more cytotoxic than either undoped or Mn-doped SPIONs. However, increasing the concentration to 600 μg mL^–1^ did not significantly amplify the cytotoxic effects of the Zn-doped SPIONs. Furthermore, at a concentration of 600 μg mL^–1^, there was no significant difference in the cell viability for any of the doped SPIONs. In this study, the cell viability for all SPIONs remained above 70% even at the maximum concentrations tested, exceeding the threshold outlined in the international standard ISO 10993–5. Zn- and Mn-doped SPIONs are commonly used doped ferrites for biomedical applications. Both metals are trace elements with high subtoxic serum concentrations of 100 and 18.3 μg dL^–1^, making them more biocompatible than other dopants such as cobalt and nickel.^44–46^ Therefore, we consider our doped ferrite nanoparticles noncytotoxic.

**Figure 3:**
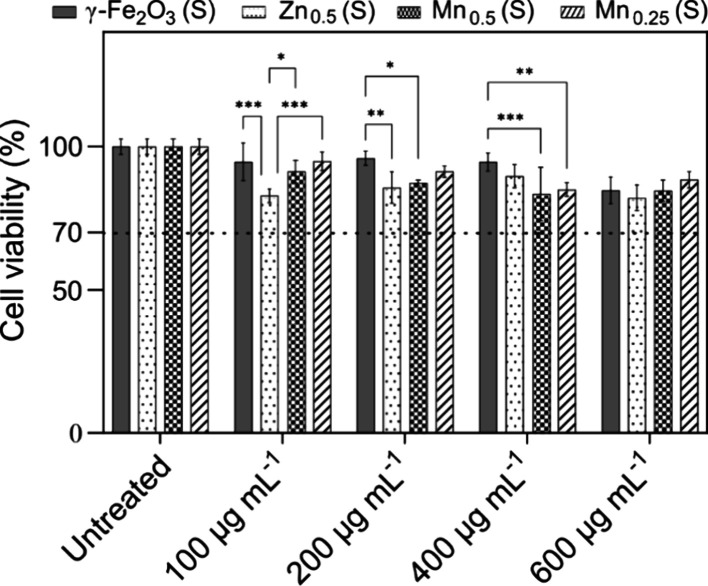
Viability of MDCK cell lines after exposure to citrate-coated suspensions of small-sized γ-Fe_2_O_3_, Zn_0.5_Fe_2.5_O_4_, Mn_0.25_Fe_2.75_O_4_, and Mn_0.5_Fe_2.5_O_4_ nanoparticles at different concentrations (100, 200, 400, and 600 μg mL^–1^). Cell viability was determined using the CellTiter-Glo luminescent cell viability assay and calculated as a percentage of the control. Dashed line represents the cytotoxicity threshold as per the international standard ISO 10993–5. Data show the average of at least four experiments ± SD. A two-way analysis of variance (ANOVA) using Tukey’s multiple comparison test was used to compare groups. The *p* values were interpreted as >0.05 (ns), ≤0.05 (*), ≤0.01 (**), and ≤0.001 (***). Only *p* values of ≤0.05 are indicated.

### Effect of Citrate Coating and Filtration on the MPI Resolution

While MPI is highly sensitive, improving tracer resolution could enable novel applications and facilitate clinical translation.^5^ Therefore, the initial assessment of MPI performance of SPIONs was based on the fwhm of the PSF derived from relaxometry measurements (Table 2), which is indicative of the expected resolution in MPI. Figure 4a shows the impact of coating and filtration on the fwhm of MPI tracers. Citrate coating and filtration improved the fwhm of large-sized undoped and Mn-doped SPIONs, which may be explained by decreased magnetic interactions between particles and the removal of large aggregates. A strong positive correlation (*R* ^2^ = 0.96) was observed between the change in the fwhm of tracers and the change in their hydrodynamic size (Figure S2). Interestingly, while the hydrodynamic size of large-sized zinc ferrite tracers changed significantly following filtration, their fwhm did not show any appreciable change. This discrepancy between large-sized Zn ferrite and other nanoparticles warrants further investigation through magnetic measurements. Peak asymmetry and shoulder were observed in PSFs of all tracers, suggesting relaxation effects. Figure 4b,c show the normalized PSFs of the small and large Mn_0.25_Fe_2.75_O_4_ nanoparticles, respectively. The small-sized tracers do not show any major difference in their fwhm after coating and filtration. On the other hand, large Mn_0.25_Fe_2.75_O_4_ nanoparticles demonstrated a substantial decrease in their fwhm after citrate coating and filtration.

**Table 2:** MPI Performance of FSP-Made Tracers Determined in the RELAX Mode and 2D Imaging Using Filtered Aqueous Suspensions of Citrate-Coated SPIONs^a^

SPION composition	fwhm [mT]	2D MPI resolution [mm]	2D MPI sensitivity (relative to VivoTrax+) [a.u. mg_Metal_^–1^]
γ-Fe_2_O_3_ (S)	15.89	3.22	22.14
γ-Fe_2_O_3_ (L)	17.16	3.59	14.38
Zn_0.5_Fe_2.5_O_4_ (S)	11.82	2.97	35.58
Zn_0.5_Fe_2.5_O_4_ (L)	11.83	2.93	26.07
Mn_0.25_Fe_2.75_O_4_ (S)	12.79	2.98	42.61
Mn_0.25_Fe_2.75_O_4_ (L)	12.54	3.07	41.46
Mn_0.5_Fe_2.5_O_4_ (S)	12.99		
Mn_0.5_Fe_2.5_O_4_ (L)	12.76		
Ferucarbotran	12.6	2.98	17.96
VivoTrax+	7.25	2.60	100
VivoTrax^51^	10.36	2.68	40.66

a MPI performance of commercial tracers is also provided for comparison.

**Figure 4:**
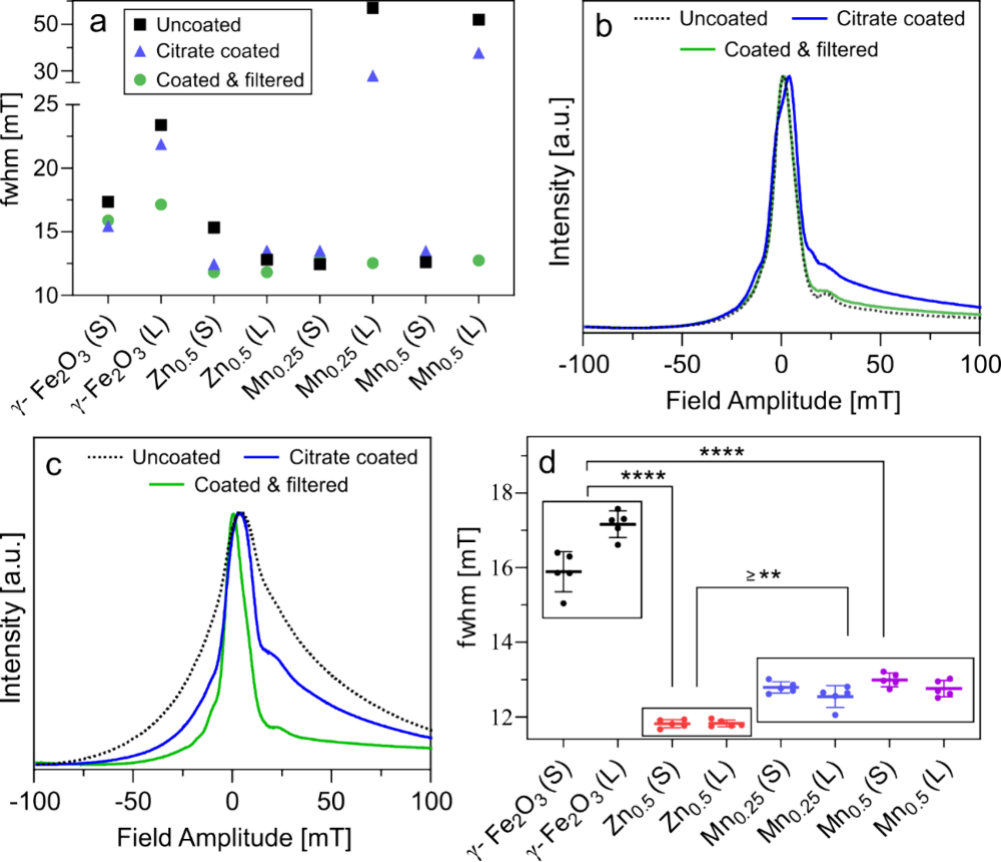
(a) Effect of citrate coating and filtration on the fwhm of the MPI tracers. *n* (number of samples) = 1 for uncoated and citrate-coated SPIONs, *n* = 5 for coated and filtered SPIONs. (b,c) Representative PSFs of uncoated, citrate-coated, and coated and filtered Mn_0.25_Fe_2.75_O_4_ nanoparticles for small (b) and large (c) crystal sizes. (d) Variance in fwhm of coated and filtered SPIONs (*n* = 5). A one-way analysis of variance (ANOVA) using Tukey’s multiple comparison test was used to compare the fwhm of coated and filtered SPIONs. The*p values were interpreted as* >0.05 (ns), ≤0.05 (*), ≤0.01 (**), ≤0.001 (***), and ≤0.0001 (****). Please note that the *y*-axis scale of Figure a differs from that of Figure d.

Previous studies have shown a strong correlation between the size of single core nanoparticles and their MPI performance, with monodisperse suspensions demonstrating superior performance than polydisperse ones.^47,48^ Similarly, fractionation of SPION suspensions to isolate an optimal size range has shown to enhance the MPI performance by up to a factor of 2.^49,50^ However, monodisperse particles can be costly to manufacture on a large scale. In this context, FSP-made SPIONs, which are subsequently processed through citrate coating and filtration, provide a cost-effective alternative.

It is crucial to note that MPI relaxometry measurements can exhibit variability. To address this, five consecutive relaxometry measurements were conducted to compare the fwhm values for each filtered tracer and assess their variance. Figure 4d presents the statistical analysis of the fwhm values for these measurements. Small and large γ-Fe_2_O_3_ particle suspensions showed the highest standard deviations of 0.54 and 0.36 mT, respectively. In contrast, the small and large Zn-doped SPIONs showed the lowest standard deviation of 0.1 mT. This discrepancy could not be explained by the degree of polydispersity of the suspensions, as the PDI of small-sized γ-Fe_2_O_3_ was lower than that of both small- and large-sized Zn ferrites (Table 1).

The average fwhm of Zn-doped SPIONs was significantly lower than those of the γ-Fe_2_O_3_ and Mn-doped SPIONs, indicating potential superior resolution of doped SPIONs (Figure 4d, Table 2). This finding aligns with a previous report where Zn_0.4_Fe_2.6_O_4_ nanoparticles exhibited a markedly reduced fwhm compared to Fe_3_O_4_ nanoparticles.^10^ Similarly, in another study, ZnFe_2_O_4_ nanoparticles showed lower fwhm than VivoTrax, a commercial tracer composed of Fe_3_O_4_.^16^ In our study, all Mn-doped ferrite tracers also consistently showed significantly lower fwhm values than those of undoped SPIONs, regardless of composition. This contrasts with findings by Dogan et al., where Mn doping led to an increase in fwhm.^11^ The discrepancy may be attributed to the differences in synthesis methods, particle compositions, particle size distributions, or the presence of weakly magnetic core–shell particles, all of which can impact the magnetic relaxation dynamics and MPI resolution.

An ANOVA test on the whole data set revealed no significant difference in the fwhm values within the γ-Fe_2_O_3_, Mn-doped, and Zn-doped SPIONs (Figure 4d). This suggests that MPI resolution was comparable between small and large particles of the same composition. These results are in disagreement with the predictions of the Langevin model, which suggests that larger particles should have greater signal strength and better resolution.^27^ The discrepancy could be due to notable deviations from the assumptions of the models, such as the effects of shape and crystal anisotropy, and particle aggregation of large flame-made SPIONs.

Consequently, the data set was categorized into three groups: γ-Fe_2_O_3_, Zn_0.5_Fe_2.5_O_4_, and Mn-doped SPIONs. To further evaluate the sensitivity and resolution, two SPION samples from each group underwent a comprehensive analysis with an iron quantification assay, dynamic magnetic susceptibility, magnetometry, and 2D MPI measurement. Specifically, small and large-sized γ-Fe_2_O_3_, Zn_0.5_Fe_2.5_O_4_, and Mn_0.25_Fe_2.75_O_4_ nanoparticles were examined. Mn_0.5_Fe_2.5_O_4_ nanoparticles were excluded from further investigations due to identical MPI resolution and poorer toxicity profile compared to those of Mn_0.25_Fe_2.75_O_4_.

### Magnetic Characterization of Doped Ferrite Tracers

Development of new MPI tracers is often guided by the Langevin equation, which describes the relationship between the MPI signal and magnetic properties of the tracers. In an idealized system, the Langevin model can be used to represent the particle response, where the peak intensity (*I*) and fwhm of the PSF can be obtained as the derivative of magnetization with respect to the applied field.^6,52^ The intensity is then given by

$$I=\frac{NM_{s}\pi D^{3}}{18}$$ 1

and the fwhm by

$$\mathrm{fwhm}=\frac{24k_{B}T}{\mu_{0}\pi GM_{s}D^{3}}$$ 2

where *N* is the number density of magnetic nanoparticles, *M*_s_ is the saturation magnetization, *D* is the nanoparticle diameter, *k*_B_ is Boltzmann's constant, *T* is the temperature, μ_0_ is the vacuum permeability, and *G* is the magnetic field gradient strength. Based on eqs 1 and 2 MPI sensitivity and resolution can be enhanced by increasing the intensity and reducing the fwhm, respectively. These improvements can be achieved by increasing the saturation magnetization and nanoparticle diameter. To assess these predictions and explore the effect of other nanoparticle properties on MPI performance, we characterized the magnetic behavior of the as-synthesized, uncoated nanoparticle powders using vibrating sample magnetometry (VSM) (Figure 5a,b). Magnetization values were normalized to the total metal content, as determined by inductively coupled plasma optical emission spectroscopy (ICP-OES).

**Figure 5:**
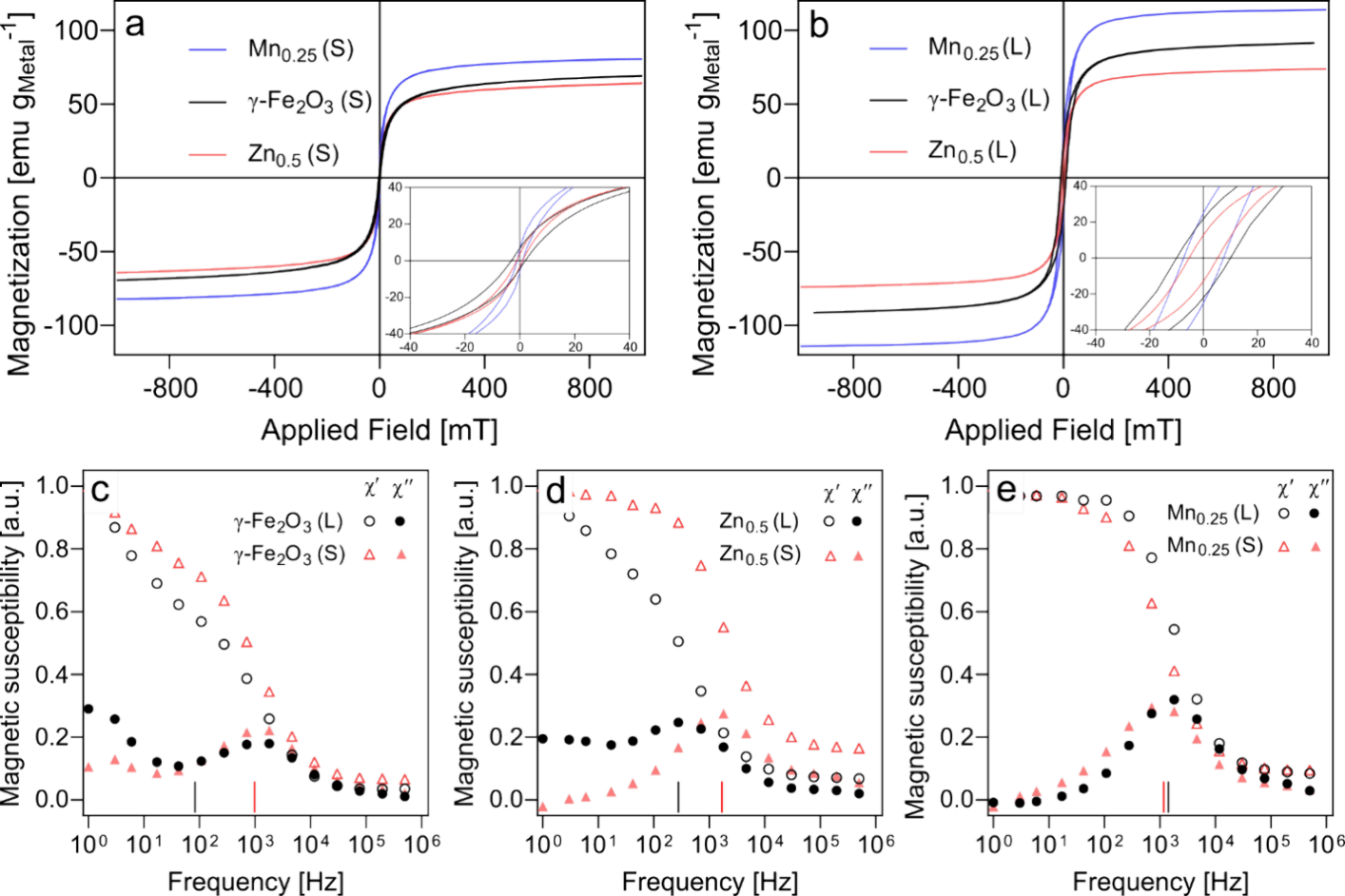
Magnetic characterization of nanoparticles at room temperature. (a, b) Magnetization vs magnetic field curves of dry powders of γ-Fe_2_O_3_, Zn_0.5_Fe_2.5_O_4_, and Mn_0.25_Fe_2.75_O_4_ for small (a) and large (b) crystal sizes. The magnetization values are normalized to the total metal content in the SPIONs. Insets show magnetization curves at ±40 mT. (c–e) Dynamic magnetic susceptibility spectra of aqueous suspensions of γ-Fe_2_O_3_ (c), Zn_0.5_Fe_2.5_O_4_ (d), and Mn_0.25_Fe_2.75_O_4_ (e) nanoparticles. The measurements were performed using 200 μL of filtered tracer suspensions, and the spectra are normalized to the low-field value of the real part χ_0_. Solid lines denote the peak frequency position for each particle calculated by using their hydrodynamic diameter, assuming that Brownian relaxation is dominant.

Most SPIONs showed negligible hysteresis (*H*_c_ < 10 mT), indicating their superparamagnetic nature (Table S2). However, large-sized γ-Fe_2_O_3_ nanoparticles displayed significant hysteresis, suggesting the presence of blocked nanoparticle magnetic moments, characteristic of a ferrimagnetic state (Figure 5a). Magnetization curves showed that all large-sized SPIONs exhibited a higher saturation magnetization than their smaller counterparts (Figure 5a,b), with values approaching those of bulk maghemite (105 emu g^–1^) and magnetite (127 emu g^–1^).^53,54^ This increase is primarily attributed to the greater number of magnetic domains in larger particles, contributing to the total magnetic moment of the material. Furthermore, in both small and large nanoparticles, Mn_0.25_Fe_2.75_O_4_ exhibited higher saturation magnetization than Zn_0.5_Fe_2.5_O_4_, which can be attributed to the higher magnetic moment of Mn^2+^ compared to Zn^2+^.^21^ This observation aligns with literature reports showing that, for a given particle size of a spinel ferrite (M_*x*_Fe_3–*x*_O_4_, M = Mn or Zn), a Zn content in the range of 0.3 < *x* < 0.5 and an Mn content ≤0.5 maximizes the measured saturation magnetization.^55–57^

To evaluate the relationship between the magnetic properties of SPIONs and their MPI resolution, we assessed how saturation magnetization, coercivity, and susceptibility influenced the fwhm (Figure S3). Negligible correlation was observed between the saturation magnetization or coercivity and fwhm (Figure S3a,b). Small-sized Zn-doped SPIONs, with relatively lower saturation magnetization, displayed the most favorable fwhm. This finding contradicts eqs 1 and 2 but aligns with observations of Velazquez-Albino et al., who have reported that the MPI resolution of tracers can correlate poorly with saturation magnetization, suggesting the need to consider other magnetic properties to explain particle performance in MPI.^6^ The fwhm showed a negative correlation (*R* ^2^ = 0.67) with the initial susceptibility, indicating that the resolution of the tracers improves with an increase in their susceptibility (Figure S3c). This agrees with reports showing a link between a steeper magnetization curve and a narrow PSF.^26^ Once again, the large-sized Zn-doped SPIONs exhibited deviations from the expected trend, showing a reduced fwhm despite their low susceptibility.

Through the magnetization curves, the impact of particle size and composition on magnetic properties was assessed under quasistatic magnetic fields. However, MPI uses oscillating magnetic fields at significantly higher frequencies, which substantially alter the magnetic response due to dipole relaxation mechanisms. Furthermore, eqs 1 and 2 assume noninteracting nanoparticles and do not account for relaxation effects. Therefore, the results from VSM may not accurately capture the dynamic behavior of tracers that influence the MPI performance.

To further investigate the magnetic response of tracers, magnetic characterization of nanoparticles was conducted by using citrate-coated and filtered aqueous suspensions (Figure S4). The magnetization data were fitted with the Langevin function considering a log-normal size distribution, as suggested by Chantrell et al.^58^ The particles showed minimal hysteresis and a large initial susceptibility, suggesting a superparamagnetic nature. The large-sized SPION suspensions (Figure S4d–f) exhibit very low hysteresis compared to the dry powder nanoparticles, likely due to the removal of larger nanoparticles and aggregates during filtration. The low-field magnetization curves of the tracers are shown in Figure S5. All particles except the large-sized Zn-doped SPIONs exhibit an overlap of the low-field magnetization loop (shown as open circles) with the full-field loop (shown as triangles), suggesting consistent magnetization behavior across the field range. In contrast, the large-sized Zn-doped SPIONs (Figure S5e) exhibit a deviation between the low-field loop and the full-field loops. This divergence in the magnetic behavior from the other samples suggests interparticle interactions, magnetic coupling, or aggregation effects introduced by Zn doping.

The interparticle interactions that likely influence the magnetic properties of the large-sized Zn-doped SPIONs could also explain their distinctly low fwhm before filtration (Figure 4a). As suggested by Moor et al., the aggregates (hard-agglomerates) of Zn-doped nanoparticles formed during flame synthesis could exhibit magnetic coupling originating from their synthesis, which can persist even under additional agglomeration.^32^ Magnetic dipole–dipole couplings increase relaxation effects,^59,60^ which in turn could influence sensitivity and image resolution in MPI.^61–63^

The volume-weighed mean magnetic diameter (*d*_M_) was calculated from the magnetization curves (Table S2). All small-sized particles closely matched their magnetic diameter with crystal size, indicating a negligible spin-canting layer and highlighting the ability of FSP to synthesize SPIONs with high crystallinity. The *d*_M_ of large-sized γ-Fe_2_O_3_ tracers also correlated well with crystal size. However, the values for the large-sized Mn-doped and Zn-doped tracers deviated considerably, with *d*_M_ values of 18.8 ± 0.5 and 12.8 ± 2 nm, compared to *d*_XRD_ values of 32.9 and 21.1 nm, respectively. The lower magnetic diameter than the crystal size could be due to the removal of a greater fraction of larger nanoparticles during filtration, bimodal magnetic size distribution, or due to the effect of multiple magnetic domains that are more common in larger crystals.^64–66^

Dynamic magnetic susceptibility measurements in Figure 5c–e show the real and imaginary components as functions of frequency. In the presence of time-varying magnetic fields, magnetic nanoparticles exhibit a response through either internal dipole rotation (Néel relaxation) or physical rotation of the particles (Brownian relaxation). The frequency-dependent characterization of SPIONs allows understanding of the particle relaxation mechanism at the excitation frequencies used in MPI.^3^ The Brownian relaxation time (τ_B_) is given by

$$\tau_{B}=\frac{3\eta V_{h}}{k_{B}T}$$ 3

where η is the viscosity of the carrier fluid (η_water_ = 10^–3^ Pa·s), *V*_h_ is the hydrodynamic volume of the nanoparticle, *k*_B_ is Boltzmann’s constant, and *T* is the temperature at which the measurement was performed (300 K).

The corresponding frequencies (*f* = 1/2πτ_B_) calculated from eq 3 were in good agreement with the observed peaks in the imaginary component (χ”) (Figure 5c–e). The peaks observed for Zn- and Mn-doped SPIONs can, therefore, be attributed to the Brownian relaxation of particles in the aqueous suspensions. This suggests the presence of magnetically blocked along with superparamagnetic particles, typical for flame-made γ-Fe_2_O_3_ nanoparticles.^67^ The peaks corresponding to Néel relaxation (τ_N_) are likely at frequencies higher than what can be measured by the instrument, such that τ_B_ > τ_N_. The Brownian relaxation time decreases after doping for both small and large SPIONs (Figure 5c–e), likely contributing to the lower fwhm of doped ferrite nanoparticles compared to undoped SPIONs (Figure 4d).^16^

### Evaluation of MPI Sensitivity and Resolution of Ferrite Tracers

Most studies on the development of doped MPI tracers use magnetic particle relaxometry or magnetic particle spectrometry.^10,11,16,17,68^ However, these methods offer only an approximation of the expected MPI performance due to differences in the imaging conditions the tracers experience and the reconstruction processes required in MPI.^7^ Furthermore, while several of these studies provide valuable insights into signal intensity, they offer limited discussion of the resolution capabilities of the tracers. A real-time assessment using an actual MPI scanner is frequently absent from these investigations. In our study, we systematically evaluated the MPI performance with 2D scans from the MOMENTUM MPI scanner, allowing measurement of spatial resolution and sensitivity of the tracers under actual scan conditions. These measurements were conducted using capillaries with an inner diameter of 1.5 mm to generate a point source of signal with a uniform cross section.^69^ Furthermore, the use of commercial tracers, such as ferucarbotran and VivoTrax+, as benchmarks allows for reproducible comparisons across studies, enabling evaluation on different instruments and settings.

The MPI signals of both small and large tracers (Figure 6a,b) were normalized to the total metal content. MPI sensitivity and resolution were determined from the maximum intensity and fwhm of the peaks, respectively. The small-sized tracers demonstrated higher sensitivity than their large-sized counterparts (Table 2), likely due to the greater magnetic susceptibility of small-sized SPIONs (Table S2). Among both small- and large-sized tracers, Mn-ferrite exhibited the highest sensitivity, followed by Zn-ferrite and γ-Fe_2_O_3_ (Figure 6a,b). This trend is in contrast with findings from a previous study, which reported relatively poor sensitivity for Mn ferrite compared to magnetite.^70^ Interestingly, in our work, Zn-ferrite tracers exhibited lower saturation magnetization and a smaller magnetic core than the undoped SPIONs (Table S2), they generated markedly higher MPI signal intensity (Figure 6a,b). A similar observation was reported by Jiang et al., where carbon-supported Zn-ferrite nanoparticles outperformed Fe_3_O_4_ despite showing lower saturation magnetization.^71^ This deviation from the predictions of the Langevin model suggests that factors beyond magnetic diameter and saturation magnetization may significantly influence MPI signal generation. The enhancement in MPI signal intensity upon incorporation of Zn into iron oxide nanoparticles is also consistent with earlier studies based on magnetic particle relaxometry.^17,68^

**Figure 6:**
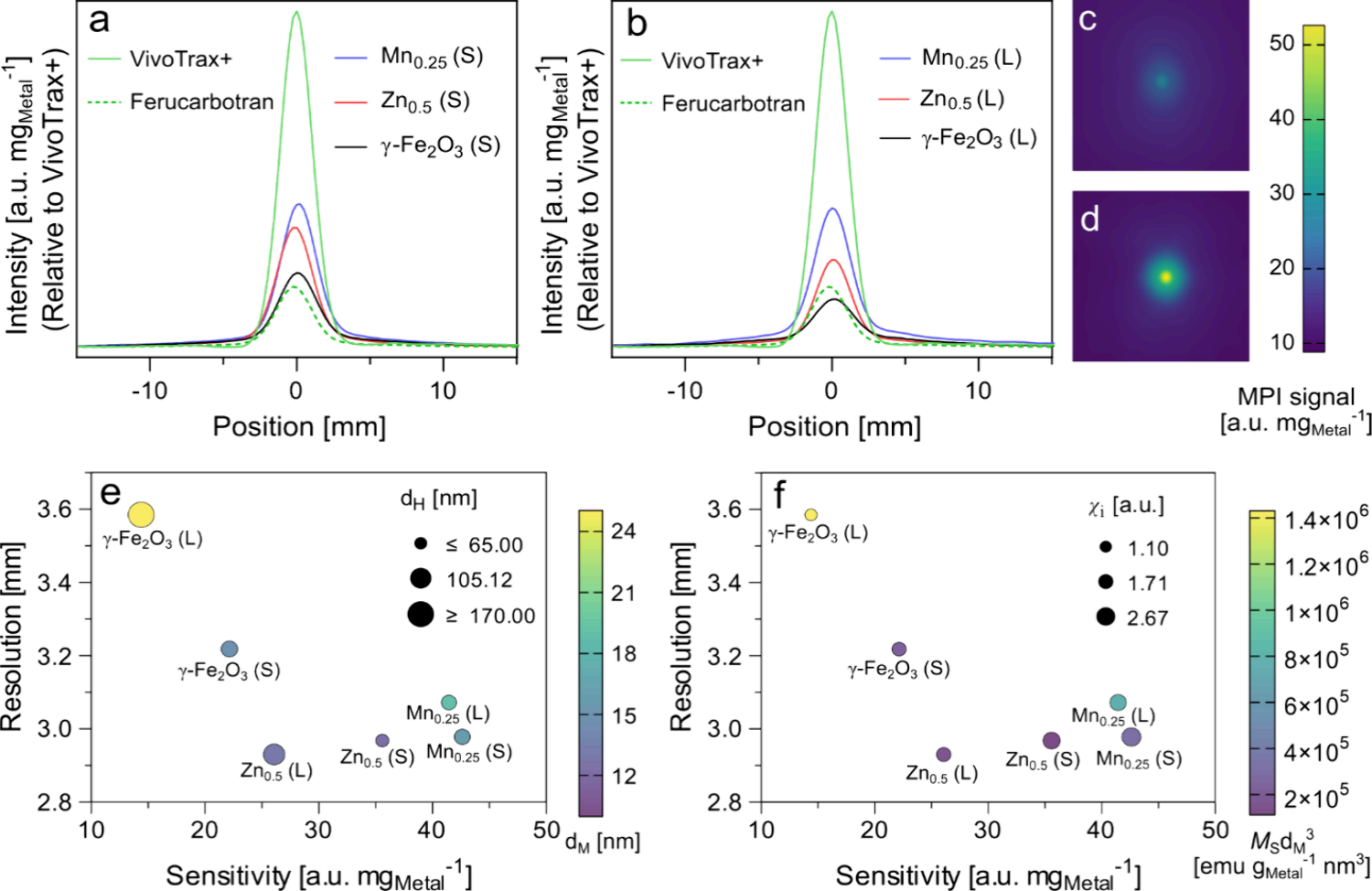
Point spread functions derived from 2D scans of (a) small- and (b) large-sized tracers. 2D MPI scans of (c) large-sized γ-Fe_2_O_3_ tracer, and (d) large-sized Zn_0.5_Fe_2.5_O_4_ tracer. Multivariate plots of MPI resolution and intensity as a function of: (e) magnetic diameter (*d*_M_, fill color) and hydrodynamic diameter (*d*_H_, marker size); and (f) saturation magnetization (*M*_s_) × *d*_M_^3^ (fill color) and initial susceptibility (χ_i_, marker size).

The MPI performance of FSP-made tracers was also compared to those of two commercially available tracers, ferucarbotran and VivoTrax+. VivoTrax+ is an improved MPI tracer derived from VivoTrax–originally developed as an MRI contrast agent–by filtering out the smaller-sized nanoparticles.^72^ The undoped SPIONs showed sensitivity comparable to that of ferucarbotran, while doped tracers exhibited higher sensitivity overall (Figure 6a,b). Although Mn-doped SPIONs showed the highest signal intensity among all synthesized tracers and were comparable to the reported intensity of VivoTrax,^51^ their sensitivity remained considerably lower than that of VivoTrax+ (Table 2, Figure 6a,b). This discrepancy can be partially explained by the differences in the magnetic diameters of the tracers. As described by eq 1, the signal intensity of a tracer is proportional to the product of its saturation magnetization and the cube of its magnetic diameter. While the reported saturation magnetization of VivoTrax+ is comparable to that of large Mn-doped SPIONs,^51^ the magnetic diameter of VivoTrax+ is larger than that of any FSP-made tracers evaluated in this study (Table S2). This larger magnetic core likely accounts for the superior sensitivity of VivoTrax+. The MPI sensitivity of FSP-made SPIONs could potentially be enhanced through improved coating strategies or methods that increase the effective magnetic diameter.

The large-sized γ-Fe_2_O_3_ SPIONs had a resolution of 3.6 mm (Figure 6c), the poorest among the synthesized tracers. Figure 6d illustrates the 2D scan of the large-sized Zn-doped SPIONs, which had the best resolution (2.93 mm) of the synthesized tracers. The difference in resolutions between large-sized Zn_0.5_Fe_2.5_O_4_ and VivoTrax+ was only 0.2 mm (Table 2), which is practically negligible for the current MOMENTUM scanner, as the default pixel size (0.25 mm) exceeds the small variation in the resolutions. This similarity in resolution between VivoTrax+ and Zn-doped SPIONs contradicts the expected performance based on the Langevin model, given their different core sizes. As discussed earlier, other factors not captured in the Langevin model could contribute to differences in tracer performance. Therefore, further studies relating particle properties to MPI performance are necessary to fully understand these effects. Nonetheless, these findings underscore the potential of FSP-made Zn and Mn ferrite nanoparticles as MPI tracers, offering high sensitivity and resolution.

The MPI performance of tracers exhibited a strong relationship with their physical and magnetic properties (Figures S6 and S7). A moderate positive correlation was observed between the MPI resolution and hydrodynamic size of the tracers (*R* ^2^ = 0.58) (Figure S6a). MPI resolution is strongly correlated to the magnetic diameter (*R* ^2^ = 0.74) and the cube of the magnetic diameter (*R* ^2^ = 0.81) of the tracers (Figure S6b,d). This is in contradiction to eq 2 which suggests an inverse correlation between magnetic diameter and resolution. Susceptibility of the tracers was negatively correlated (*R* ^2^ = 0.51) with the MPI resolution (Figure S6c), indicating that particles with the sharpest magnetization response had the best resolution. Furthermore, MPI resolution worsened with an increase of the product of saturation magnetization and the cube of magnetic diameter (*R* ^2^ = 0.72), also in contradiction to eq 2 (Figure S6e). On the other hand, MPI sensitivity was moderately correlated with hydrodynamic size (*R* ^2^ = 0.62) and very strongly correlated with susceptibility (*R* ^2^ = 0.88) (Figure S7a,c). Interestingly, sensitivity showed a poor correlation with other magnetic characteristics of the tracers (Figure S7), which contradicts eq 1, where the intensity of the tracer is proportional to the cube of magnetic diameter. Overall, these findings agree with previous studies demonstrating the limitations of the Langevin model in accurately predicting the variability in MPI performance of tracers with different physical and magnetic properties.^6,68^

The overall effect of tracer properties on MPI performance is shown in Figure 6e,f. Tracers with a smaller hydrodynamic size had better resolution and sensitivity (Figure 6e), indicating that reducing the tracer hydrodynamic size through citrate coating and filtration improves the MPI performance. Improving both the magnetic diameter and saturation magnetization could further enhance MPI performance (Figure 6e,f). Magnetic diameter can be optimized through the careful control of the FSP parameters to minimize the spin-canting layer and maintain the average size below the superparamagnetic regime. Tracers with a large susceptibility showed better MPI performance (Figure 6e), suggesting that incorporating dopants in SPIONs to yield higher susceptibility could be a straightforward way to improve the MPI resolution and sensitivity. However, the weak trends between the magnetic properties and MPI sensitivity (Figure S7) support the need to account for other particle characteristics that influence tracer performance.

Overall, these findings highlight that the magnetic susceptibility and hydrodynamic size are among the key drivers of tracer sensitivity. However, improvement in resolution is less straightforward and likely influenced by additional factors, such as polydispersity, magnetic core size, and particle interactions. These results, along with those of Velazquez-Albino et al. and Imhoff et al., indicate that the relationship between MPI performance and the intrinsic physical and magnetic properties of tracers is likely more intricate than what can be accounted for by the Langevin model.^6,51^ Nonetheless, overall, these findings underscore the ability of FSP to finely tailor SPION composition and size, offering the potential for improved MPI performance and high scalability.

### Scalable Formulation of Aqueous Dispersion of SPIONs Using Flash Nanoprecipitation

The synthesis of undoped and doped SPIONs using FSP shows its applicability for the scalable production of MPI tracers. However, the multistep, postsynthesis process of citrate coating and filtration could be a hindrance to efficient scale-up of MPI tracer production.^73,74^ Therefore, flash nanoprecipitation (FNP) was used as a complementary, scalable technique to transfer the dry SPION powders into aqueous suspensions (Figure 1). In FNP, nanocarriers are produced by encapsulating SPIONs in poly­(lactic acid)-*b*-polyethylene glycol, a stabilizing amphiphilic block copolymer. This is achieved through rapid mixing of a water-miscible organic solvent such as dimethyl sulfoxide (DMSO), containing nanoparticles and copolymer, with water as an antisolvent.^75^ Previous studies have used FNP for the development of nanocarriers for MPI and MRI.^76,77^ The results presented here are a proof-of-principle of the rapid and efficient transfer of flame-made SPIONs into water. Figure 1 illustrates the multi-inlet vortex mixer used for the FNP process, as described elsewhere.^75^ The small-sized Zn_0.5_Fe_2.5_O_4_ nanoparticles were selected for the fabrication of magnetic nanocarriers due to their consistent demonstration of high MPI resolution and sensitivity, as well as excellent dispersibility in DMSO.

The nanocarriers produced by FNP showed a larger hydrodynamic size (148 nm) than citrate-coated small-sized Zn_0.5_Fe_2.5_O_4_ nanoparticles (65 nm), as would be expected from encapsulating multiple nanoparticles into a single polymeric nanocarrier. Figure 7 shows the signal intensity profile of the magnetic nanocarrier and citrate-coated SPIONs. The nanocarriers showed a 30% increase in MPI sensitivity compared to the citrate-coated Zn-doped nanoparticles, which could be due to reduced particle aggregation in nanocarriers resulting from the encapsulation of SPIONs in the amphiphilic polymer. Furthermore, the nanocarriers achieved a resolution of 3.2 mm, closely matching that of the citrate-coated Zn-doped SPIONs (2.96 mm). These nanocarriers maintained their size when stored at 4 °C, measuring 150 nm after 5 days (Figure 7 inset). These findings highlight FNP as an alternative to the multistep coating and filtration process. Integrating FNP with FSP established a streamlined and scalable workflow for generating water-dispersible MPI tracers, enhancing the translational potential of doped ferrite tracers for both research and clinical applications. FNP also offers the potential to incorporate drugs for the development of theranostic systems.^75^

**Figure 7:**
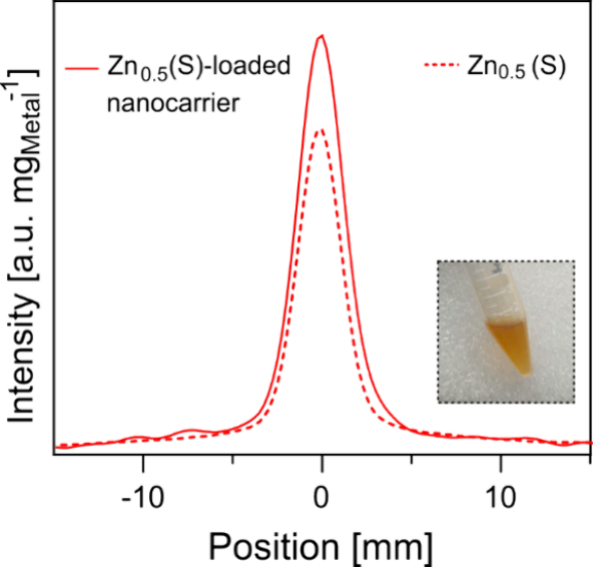
PSF derived from 2D MPI scans of nanocarriers produced by flash nanoprecipitation (solid line) incorporating small-sized Zn_0.5_Fe_2.5_O_4_ nanoparticles, and the citrate coated small-sized Zn_0.5_Fe_2.5_O_4_ nanoparticles (dashed line). The inset shows the nanocarrier suspension after 5 days of storage at 4 °C.

## Conclusions

This study investigates the development of Zn- and Mn-doped SPIONs as MPI tracers. The SPIONs were synthesized using FSP, which enabled precise control over particle size and composition, allowing us to systematically investigate the effect of these properties on the MPI performance. Citrate coating and filtration effectively reduced the polydispersity and hydrodynamic size of tracers, enhancing both resolution and sensitivity. The doped tracers demonstrated improved MPI performance compared to the undoped nanoparticles, while the low in vitro cytotoxicity of doped ferrites indicated their biocompatibility. The 2D MPI scans showed that the large-sized Zn_0.5_Fe_2.5_O_4_ nanoparticles achieved the best resolution (2.93 mm), and the small-sized Mn_0.25_Fe_2.75_O_4_ tracers exhibited the highest sensitivity among the FSP-made ferrites. While the MPI performance of the FSP-made doped ferrites was better than that of ferucarbotran, the sensitivity of VivoTrax+ remained higher than that of the FSP-made tracers. However, the negligible resolution difference between VivoTrax+ and Zn ferrite nanoparticles highlights the potential of FSP to produce high-quality MPI tracers. Our findings show that while both MPI sensitivity and resolution are influenced by the hydrodynamic size and magnetic susceptibility of SPIONs, the resolution is additionally affected by polydispersity and magnetic core diameter. These observations also highlight the limitations of the Langevin model in capturing the variability of the MPI performance across tracers with diverse physical and magnetic properties. Nonetheless, the use of consistent characterization (structural, magnetic, relaxometric, and 2D imaging) and the comparison with commercial tracers offers a robust approach for future tracer development. Our findings also suggest that optimal resolution and maximum sensitivity often do not coincide, indicating that a trade-off may be needed based on the target application. Lastly, integration of FNP with FSP enabled the production of a stable, water-dispersible Zn ferrite tracer without compromising the MPI performance. The combination of FSP and FNP presents a scalable workflow that should be further explored for both therapeutic and diagnostic applications.

## Experimental Section

### Synthesis of Nanoparticles

The undoped and doped SPIONs used here were produced by FSP in our previous study.^19^ For the sake of completeness, the synthesis technique is briefly described here and also presented in Table S1. Liquid precursor solutions for undoped SPIONs were prepared by dissolving iron­(III) nitrate nonahydrate (Sigma-Aldrich, Sweden) in a solvent mixture (1:1) of 2-ethylhexanoic acid (Sigma-Aldrich) and ethanol (VWR, Belgium). Zinc- and manganese-doped SPIONs were synthesized by the addition of the dopant precursor, either zinc nitrate hexahydrate or manganese­(II) nitrate tetrahydrate (Sigma-Aldrich). The metal concentration was between 0.6 and 0.7 mol L^–1^. The precursor solutions were stirred for at least 1 h at room temperature. The pilot flame was ignited by a premixed supporting flame of CH_4_ and O_2_ (>99.5%, Linde AGA Gas AB, Sweden) at flow rates of 1.5 and 3.2 L min^–1^, respectively. The precursor was fed to the pressure-assisted nozzle (1.6 bar) with a precision syringe pump and dispersed by using O_2_. The precursor and dispersion gas flow rates were varied to obtain the desired nanoparticle size (Table S1). Sheath gas (O_2_) at 5 L min^–1^ was fed through the outermost sintered metal plate of the FSP burner. Gas flow rates were controlled with calibrated mass flow controllers (Bronkhorst, The Netherlands). The particles were collected on a glass fiber filter (Albert LabScience, Germany) with the aid of a Mink MM 1144 BV vacuum pump (Busch, Sweden).

### Citrate Coating of Nanoparticles

To perform the citrate coating, 25 mg of nanoparticle powder was dispersed in 5 mL of ultrapure water by ultrasonication using a probe sonicator (Sonics, USA) for 5 min at 35% amplitude with a pulse of 30 s on and 2 s off. Trisodium citrate dihydrate (50 mg) was added to the suspension, and the mixture was shaken for 30 s, followed by ultrasonication for another 5 min. The reaction mixture was heated at 70 °C and stirred magnetically for 30 min, after which the suspension was cooled to room temperature. The resulting product was purified from unreacted citrate by centrifugation at 6000 × *g* for 10 min. Subsequently, the supernatant was discarded, and the resulting pellet was washed twice with water. Finally, the citrate-coated ferrites were redispersed in ultrapure water at a concentration of 5 mg mL^–1^. The coated nanoparticles were filtered through a poly­(ether sulfone) membrane syringe filter (0.22 μm, Sigma-Aldrich).

### Fabrication of Magnetic Nanocarriers

Nanoparticles were suspended in dimethyl sulfoxide (DMSO) at a concentration of 3 mg mL^–1^. A 10 mg mL^–1^ solution of the block copolymer polylactic acid-*b*-polyethylene glycol (PLA-*b*-PEG; Evonik Industries, Germany) was prepared in tetrahydrofuran (Thermo Fisher Scientific, USA). Subsequently, two separate airtight syringes were loaded with 2 mL of SPION suspension and copolymer solution, while another two were loaded with 2 mL of water. These four syringes were connected to a multi-inlet vortex mixer (MIVM), which drained into a beaker containing 180 mL of ultrapure water. The syringe pistons were pushed simultaneously into the MIVM at approximately 60 mL min^–1^ using an in-house 3D-printed apparatus. For the removal of tetrahydrofuran and the composite particles without SPIONs, magnetic filtration was performed by using magnetic columns (Miltenyi Biotec, Germany).

### Characterization of Nanoparticles

Undoped and doped SPIONs used in this study were partially characterized in our previous work, where some of their properties have been reported.^19^ For the sake of completeness, all the relevant characterization techniques are briefly described here. The X-ray diffraction (XRD) patterns of dry powder nanoparticles were measured at ambient temperature on a MiniFlex X-ray diffractometer (Rigaku Europe, Germany) using Cu Kα1 radiation (1.5406 Å) at 40 kV and 15 mA. The patterns were recorded between 10 and 80° 2θ at a step size of 0.01° and a scan speed of 2.00° min^–1^. A DTEX detector was used to suppress the iron fluorescence background. The XRD data were analyzed using PDXL2 (version 2.8, Rigaku Europe). All patterns were normalized relative to the peak intensity corresponding to the (311) crystal plane. The average crystal size (*d*_XRD_) of nanoparticles was calculated by Rietveld refinement analysis and Scherrer equation using the PDXL2 software.

The specific surface area of nanoparticles was determined by nitrogen adsorption at 77 K following the Brunauer–Emmett–Teller (BET) method on a TriStar II Plus system (Micromeritics, USA) after degassing of nanoparticle powder for at least 3 h at 110 °C under a flow of nitrogen gas. Grain size (*d*_BET_) was calculated from the specific surface area (with ρ_γ‑Fe2O3_ = 4.9 g cm^–3^) by assuming spherical particles. The hydrodynamic size was measured by dynamic light scattering, with the samples diluted with deionized water to a nanoparticle concentration of 0.1 mg mL^–1^. The measurements were conducted on a ZetaPALS instrument (Brookhaven Instruments), at a scattering angle of 90° and at room temperature. The Fourier-transform infrared (FTIR) spectra were obtained from an ALPHA II spectrometer (Bruker, Germany) with a platinum ATR accessory, in the range from 400–4000 cm^–1^.

Inductively coupled plasma optical emission spectroscopy (ICP-OES) was used to quantify the iron present in the tracers. To prepare the measurement sample, 10 μL of tracer suspension was dissolved in 300 μL of 37% hydrochloric acid (Sigma-Aldrich) and heated at 80 °C for 1 h. The solution was then cooled to room temperature and analyzed by using ICP-OES.

Magnetic properties of dry nanoparticles were measured on a vibrating sample magnetometer (Lake Shore, USA). The magnetization versus magnetic field was measured in the field range ±1000 mT at room temperature. The saturation magnetization and coercivity were calculated from the obtained magnetization curves. The initial susceptibility (χ_i_) was calculated as the ratio of magnetization (*M*) to the applied magnetic field strength (*H*) at low magnetic fields (<5 mT), where the linear approximation is valid. Magnetic characterization of nanoparticle suspensions was performed on a Magnetic Property Measurement System 3 (Quantum Design Inc., USA) superconducting quantum interference device (SQUID). The measurements were performed at 300 K using 100 μL of nanoparticle suspension placed in a PTFE sample holder. Magnetic diameters (*d*_M_) of the SPIONs in suspensions were obtained by fitting the magnetization curves to the Langevin function for superparamagnetism, weighted using a log-normal size distribution,^6^ as suggested by Chantrell et al.^58^ A nonlinear regression model was used to perform the fit in MATLAB (MathWorks, USA), providing an estimate of the average magnetic diameters (assuming that the magnetic domains are spherical). The dynamic magnetic susceptibility of nanoparticle suspensions was measured using a DynoMag AC susceptometer (Rise Research Institutes, Sweden). This measurement was recorded at room temperature as a function of the frequency of the oscillating magnetic field with 200 μL of suspension using a small amplitude oscillating magnetic field.

### Cytotoxicity of Nanoparticles

A cell viability assay was performed to assess the cytotoxicity of small-sized nanoparticles. Cell culture media and reagents were purchased from Thermo Fisher Scientific or Sigma-Aldrich. Madin-Darby canine kidney cells (MDCKcMDR1-KO), passage 21, were provided by the Drug Delivery group, Dept of Pharmacy, Uppsala University.^78^ Cells were maintained in low-glucose Dulbecco’s modified Eagle’s medium with GlutaMAX, containing 10% (v/v) fetal bovine serum, and 1% (v/v) penicillin (10 mg mL^–1^)–streptomycin (10 mg mL^–1^) solution. Cells were cultured at 37 °C in a humidified incubator in 75 cm^2^ tissue culture flasks at 10% CO_2_.

SPION cytotoxicity was assessed using citrate-coated small γ-Fe_2_O_3_, Zn_0.5_Fe_2.5_O_4_, Mn_0.25_Fe_2.75_O_4_, and Mn_0.25_Fe_2.75_O_4_ nanoparticles. A stock suspension of each citrate-coated ferrite solution was prepared at 6 mg mL^–1^ as described above, then diluted with the corresponding cell culture medium to achieve nanoparticle concentrations of 600, 400, 200, and 100 μg mL^–1^. The cells were plated into black and opaque 96-well plates, at a density of 5 × 10^4^ cells per well in 250 μL of culture medium. The cells were allowed to attach to the plate for 24 h before being incubated with the citrate-coated nanoparticles. Thereafter, the cell culture media were replaced with 100 μL of nanoparticle suspensions in four replicates and incubated for 24 h. Cell culture media (without nanoparticles) were used as the untreated control, while controls for 100% cell death were prepared by incubating the cells in culture medium containing 0.2% (v/v) Triton X-100 (Sigma-Aldrich). The viability of the cells was assessed by the CellTiter-Glo Luminescence assay (Promega, USA) according to the manufacturer's instructions. The luminescence signal of each well was determined with a plate reader (Tecan, Switzerland).

### MPI Performance of Tracers

All MPI measurements were performed on a MOMENTUM preclinical scanner (Magnetic Insight). The x-space point spread function (PSF) was measured using the RELAX module of the scanner operating at a driving field with an amplitude of 16 mT and a frequency of 45 kHz. A volume of 3–50 μL of each tracer was placed in 0.2 mL microcentrifuge tubes and positioned in the center of the MPI field of view using a custom 3D-printed sample holder.^79^ Positive scans were used to plot the PSF, and the signal intensity was calculated by normalizing the PSF amplitude to the total mass of metal. The fwhm is the system-reported value without normalization. Five consecutive PSF scans were performed for the same sample, unless otherwise indicated.

For a visual comparison of the tracers’ performance in a real MPI imaging system, 2D images were acquired with the MOMENTUM scanner in “standard” multichannel scan mode using 5.7 T m^–1^ gradient strength. The measurements were conducted using excitation field strengths of 16 and 19 mT in the x-channel and z-channel, respectively, and an excitation frequency of 45 kHz. The MPI performance of flame-made SPIONs was compared to the commercially available tracers Ferucarbotran (0.5 mmol_Fe_ mL^–1^) and VivoTrax+ (5.5 mg_Fe_ mL^–1^), obtained from Meito Sangyo Co., Ltd. (Japan) and Magnetic Insight (USA), respectively. Samples consisted of 3–30 μL of suspension in a capillary tube (Ø 1.5 mm) placed parallel to the *y*-axis in the center of the MPI field of view, in custom 3D-printed holders. MPI scans of the sample holders were acquired prior to their use to measure any signal contribution from them. All capillary tube ends were sealed with a Cha-Seal to prevent sample evaporation. The images were analyzed using MATLAB (MathWorks, USA) in-house algorithms, in which a line scan was taken through the center of each image source to obtain a maximum signal intensity normalized by total metal mass and resolution calculated by the fwhm of the curve. The fwhm obtained from scans along the *z*-axis was used for comparison.

### Statistical Analysis

Data analysis was performed using GraphPad Prism 10.0 (GraphPad Software Inc., USA). Either one-way or two-way analysis of variance (ANOVA) using Tukey’s multiple comparison test was used to compare groups. The *p* values were interpreted as >0.05 (ns), ≤0.05 (*), ≤0.01 (**), ≤0.001 (***), and ≤0.0001 (****). Coefficients of determination (*R* ^2^) were determined using simple linear regressions.

## Supplementary Material



## Notes

X-ray diffraction pattern of SPIONs (Figure S1); change in fwhm of tracers as a function of the change in their hydrodynamic size (Figure S2); correlation between fwhm of tracers and their magnetic properties (Figure S3); magnetization curves of tracers in aqueous suspensions (Figure S4); magnetization curves of tracers at low field (Figure S5); relationship between MPI resolution and tracer properties (Figure S6); relationship between MPI sensitivity and tracer properties (Figure S7); summary of FSP synthesis parameters used to produce SPIONs (Table S1); and structural and magnetic properties of SPIONs (Table S2) (PDF)

## Abbreviations

FNP: flash nanoprecipitation
FSP: flame spray pyrolysis
FTIR: Fourier-transform infrared
fwhm: full-width at half-max
MIVM: multi-inlet vortex mixer
MPI: magnetic particle imaging
MRI: magnetic resonance imaging
PDI: polydispersity index
PSF: point spread function
SPION: superparamagnetic iron oxide nanoparticle
XRD: X-ray diffraction
