# Supplementary material for: Flame-Made Doped Iron Oxide Nanoparticles as Tracers for Magnetic Particle Imaging
Source: Chem Mater. 2025 May 20;37(11):4071–84. doi: 10.1021/acs.chemmater.5c00331 (PMC12159977; doi:10.1021/acs.chemmater.5c00331)
Supplement: Supplementary file 1 [file cm5c00331_si_001.pdf]

Supporting information

Flame-made doped iron oxide nanoparticles as tracers for magnetic particle imaging

Shaquib Rahman Ansari[1], Eric Daniel Imhoff[2], Yael del Carmen Suárez-López[1], Andrii Melnyk[2], Carlos M. Rinaldi-Ramos[2], and Alexandra Teleki[1*]*

[1]Department of Pharmacy, Science for Life Laboratory, Uppsala University, 75123, Uppsala, Sweden

[2]Department of Chemical Engineering and J. Crayton Pruitt Family Department of Biomedical Engineering, University of Florida, 32611-6005, Gainesville, FL, USA

*Email: alexandra.teleki@scilifelab.uu.se and carlos.rinaldi@ufl.edu

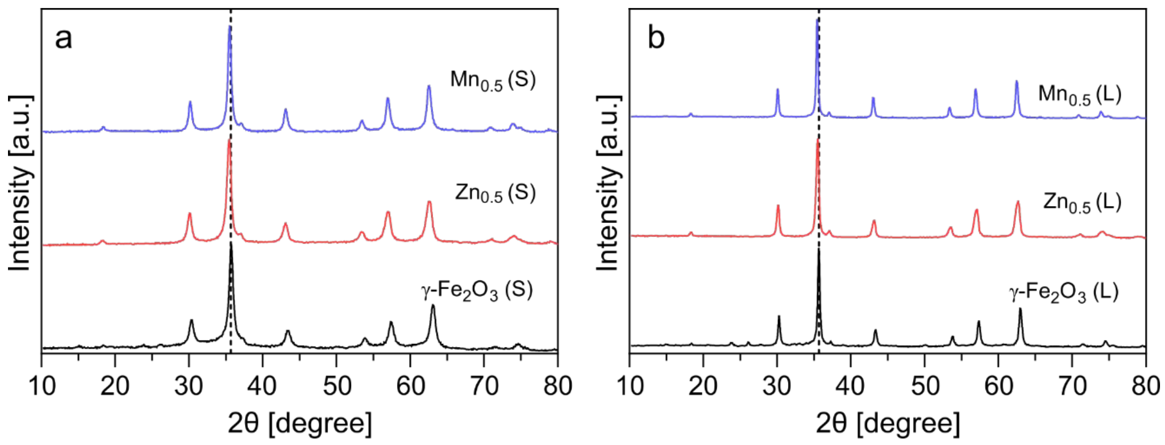

Figure S1. X-ray diffraction patterns of (a) small-sized and (b) large-sized nanoparticles. The colors indicate γ-Fe_2O_3 (black), $Zn_{0.5}Fe_{2.5}O_4$ (red), and $Mn_{0.5}Fe_{2.75}O_4$ (blue) nanoparticles. The dashed line represents the maghemite (311) peak.

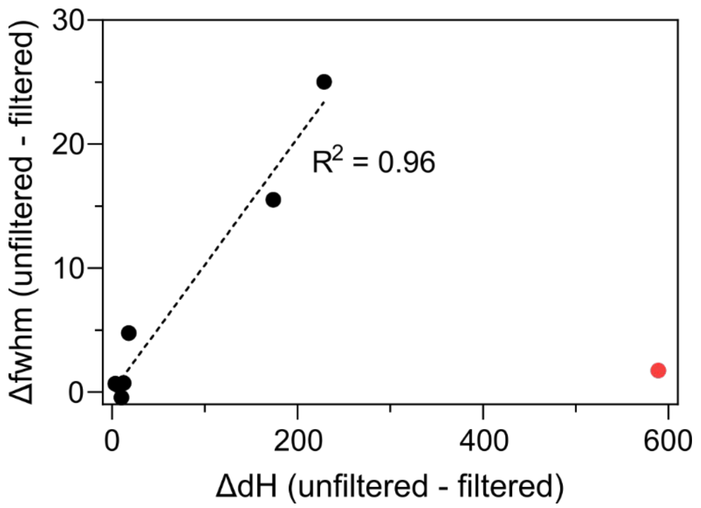

Figure S2. The change in full-width at half maximum (fwhm) of the tracers as a function of the change in their hydrodynamic size (dH). The R^2 was determined using linear regression after excluding the measurements of $Zn_{0.5}$ (L) tracer (indicated in red).

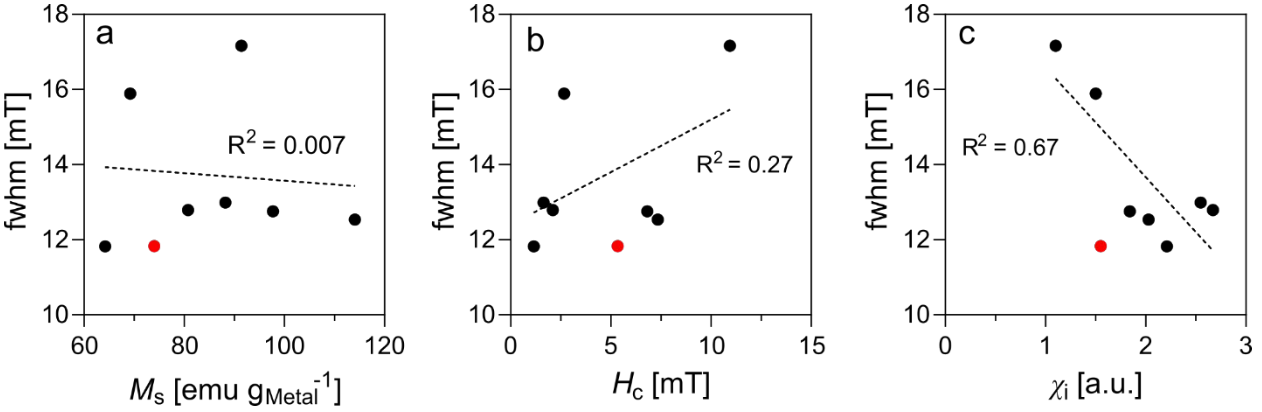

Figure S3. Correlation between fwhm of the tracers and their (a) saturation magnetization (M_s), (b) coercivity (H_c), and (c) initial susceptibility (χ_i). The magnetic parameters were measured using nanoparticle powders. The R^2 was determined using linear regression after excluding the measurements of $Zn_{0.5}$ (L) tracer (indicated in red).

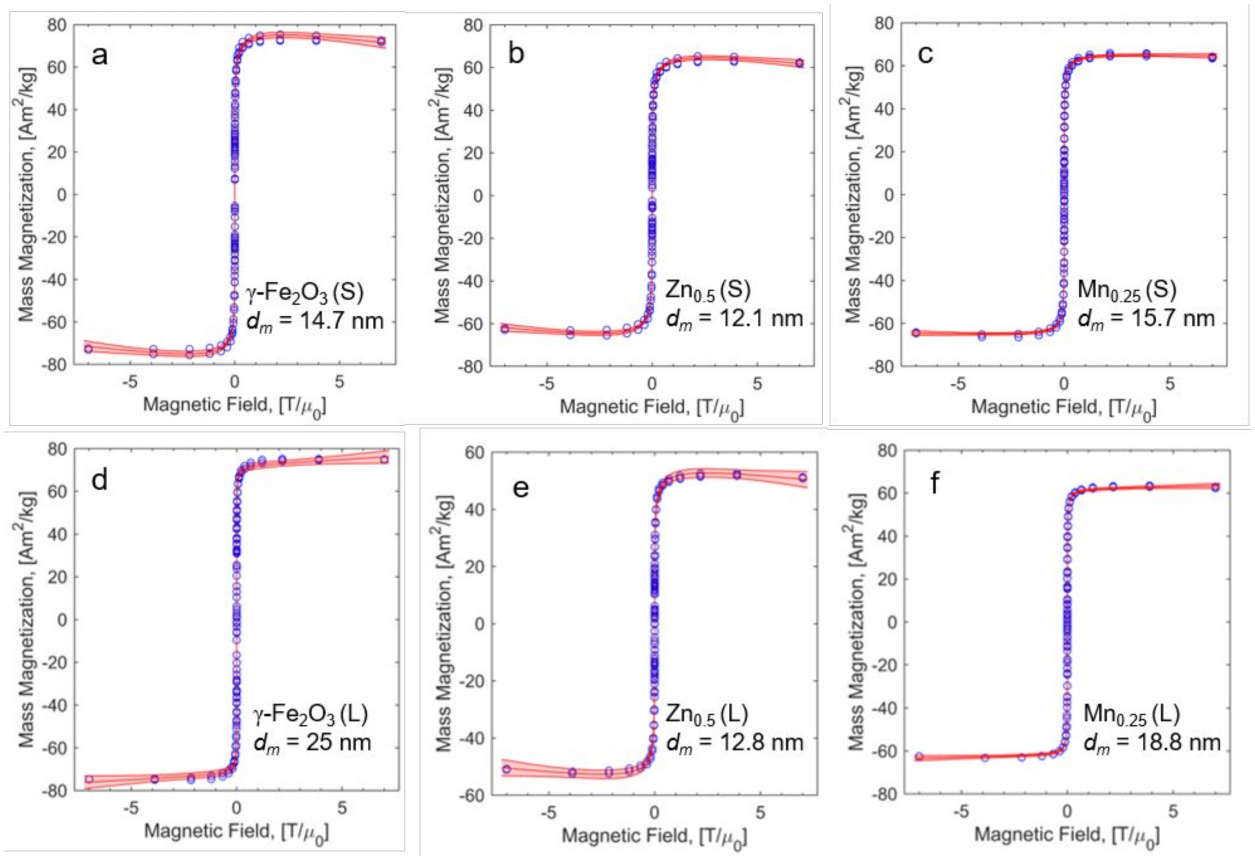

Figure S4. Magnetization curves of flame-made MPI tracers. a-c) small-sized γ-Fe_2O_3 (a), $Zn_{0.5}Fe_{2.5}O_4$ (b), and $Mn_{0.25}Fe_{2.75}O_4$ (c); d-f) large-sized γ-Fe_2O_3 (d), $Zn_{0.5}Fe_{2.5}O_4$ (d), and $Mn_{0.25}Fe_{2.75}O_4$ (e). The measurements were performed on a SQUID magnetometer at 300 K using 100 μL aqueous suspensions of SPIONs.

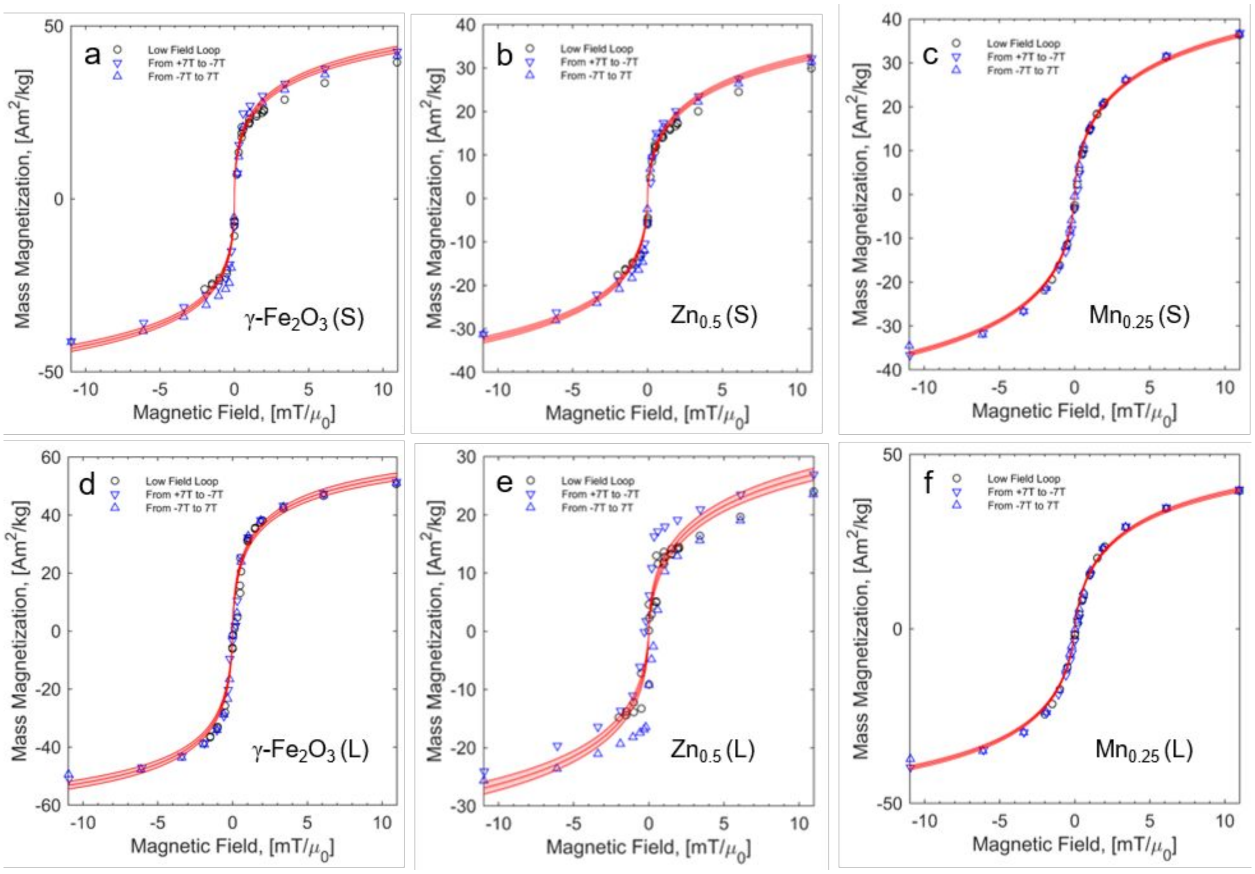

Figure S5. Magnetization curves of flame-made MPI tracers at low field (± 10 mT). a-c) small-sized γ-Fe_2O_3 (a), $Zn_{0.5}Fe_{2.5}O_4$ (b), and $Mn_{0.25}Fe_{2.75}O_4$ (c); d-f) large-sized γ-Fe_2O_3 (d), $Zn_{0.5}Fe_{2.5}O_4$ (d), and $Mn_{0.25}Fe_{2.75}O_4$ (e). The measurements were performed on a SQUID magnetometer at 300 K using 100 μL aqueous suspensions of SPIONs.

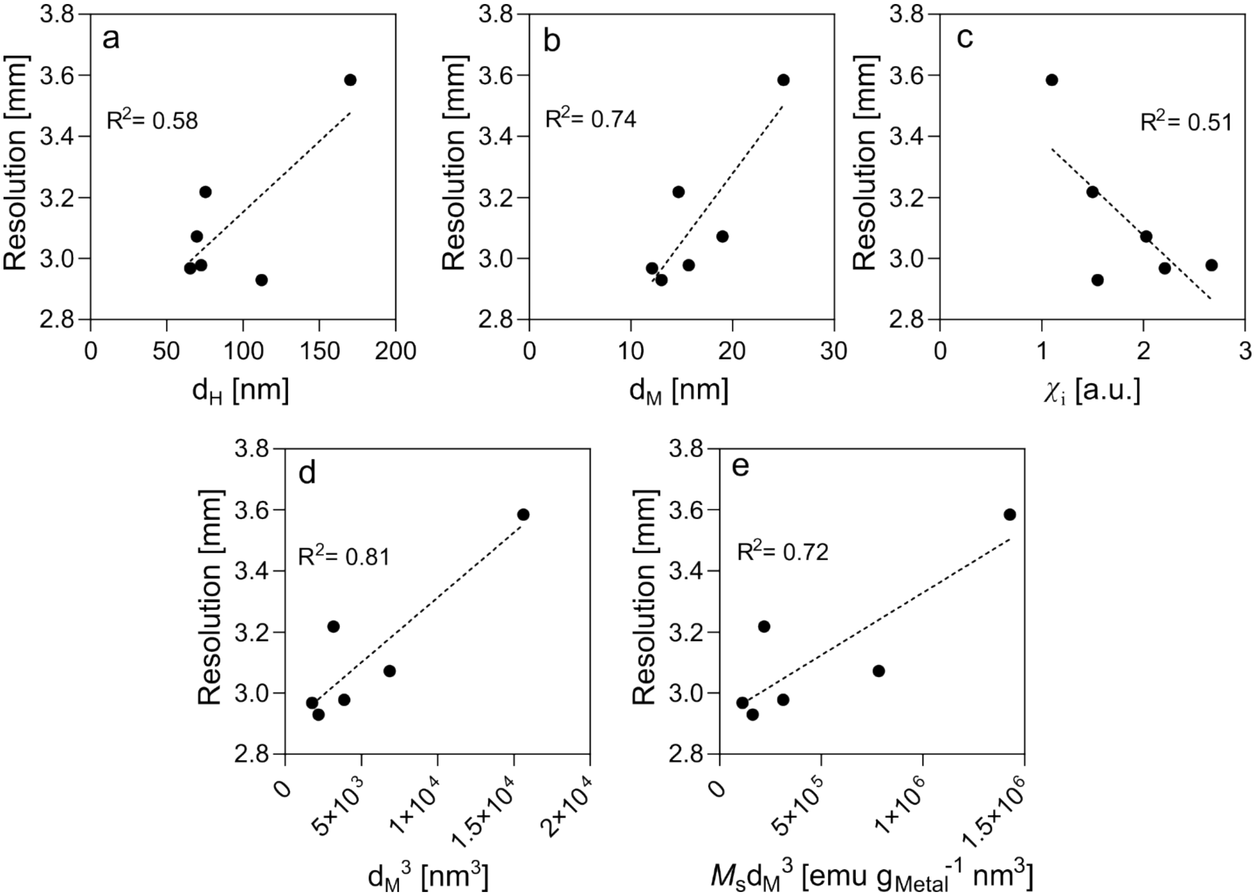

Figure S6. Relationship between MPI resolution and tracer properties: (a) hydrodynamic size (d_H), (b) magnetic diameter (d_M), (c) initial susceptibility (χ_i), (d) cube of magnetic diameter, and (e) the product of saturation magnetization (M_s) and cube of magnetic diameter.

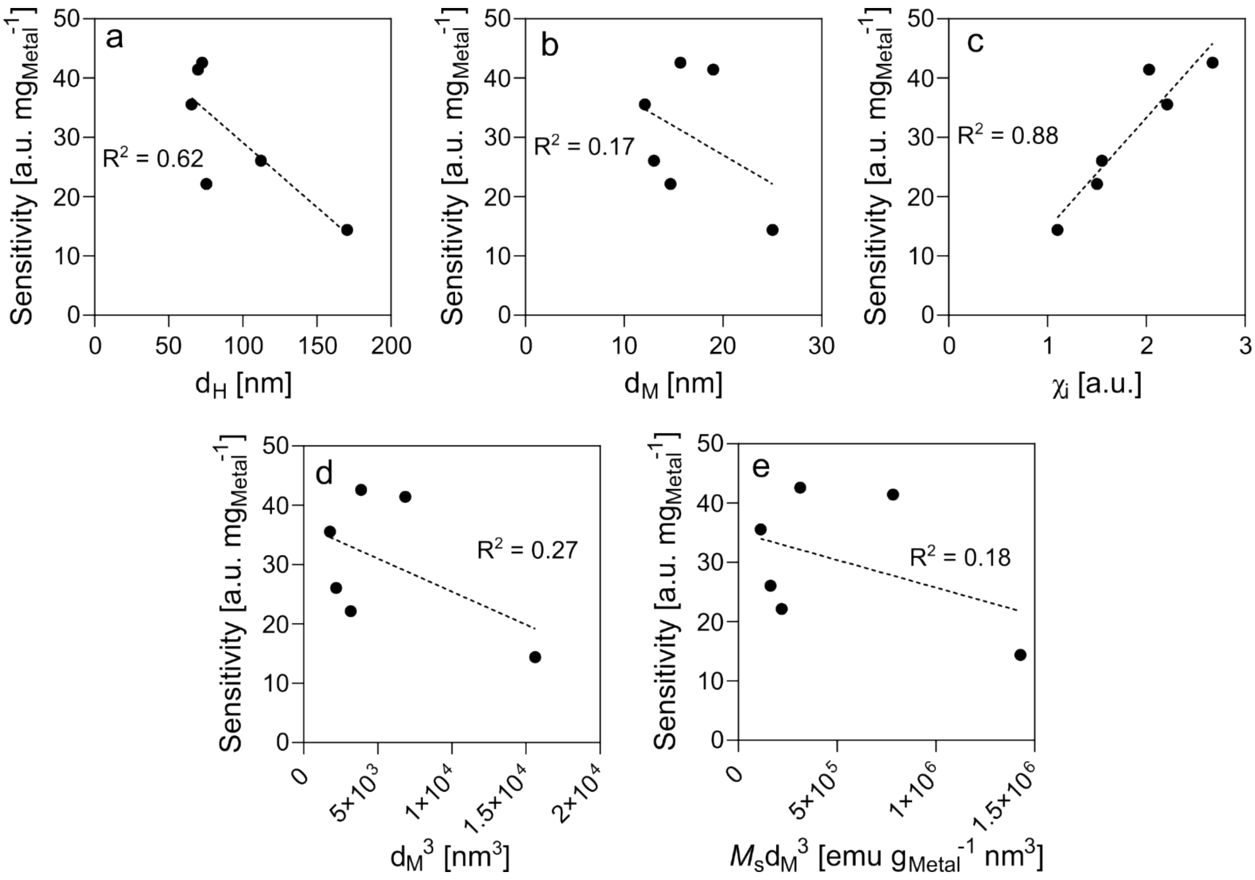

Figure S7. Relationship between MPI sensitivity and tracer properties: (a) hydrodynamic size (d_H), (b) magnetic diameter (d_M), (c) initial susceptibility (χ_i), (d) cube of magnetic diameter, and (e) the product of saturation magnetization (M_s) and cube of magnetic diameter. The sensitivity is calculated relative to VivoTrax+.

Table S1. Summary of FSP parameters used to produce SPIONs.

**SPION composition**	**Precursor concentration [mol_{Metal} L^{-1}]**	**Precursor flow rate [mL min^{-1}]**	**Dispersion gas flow rate [L min^{-1}]**
γ-Fe_2O_3 (S)	0.7	6	3
γ-Fe_2O_3 (L)	0.6	12	3
$Zn_{0.5}Fe_{2.5}O_4$ (S)	0.7	6	3
$Zn_{0.5}Fe_{2.5}O_4$ (L)	0.7	15	3
$Mn_{0.25}Fe_{2.75}O_4$ (S)	0.7	6	3
$Mn_{0.25}Fe_{2.75}O_4$ (L)	0.7	15	3
$Mn_{0.5}Fe_{2.5}O_4$ (S)	0.7	6	3
$Mn_{0.5}Fe_{2.5}O_4$ (L)	0.7	15	3

Table S2. Structural and magnetic properties of SPIONs showing particle size derived from specific surface area (d_{BET}), average crystal size (d_{XRD}), magnetic diameter (d_M), saturation magnetization (M_s), Coercivity (H_c), and initial susceptibility (χ_i). The magnetic diameter was obtained from filtered aqueous suspensions of citrate coated SPIONs, while rest of the properties were obtained from as-synthesized dry nanoparticle powder.

SPION composition	d_{BET} [nm]	d_{XRD} [nm]	d_M [nm]	M_s [emu g_{Metal}^{-1}]	H_c [mT]	χ_i [a.u.]
γ-Fe_2O_3 (S)	15.3	15.4	14.7 ± 1.8	69.2	2.7	1.5
γ-Fe_2O_3 (L)	20.7	23.9	25 ± 1.3	91.4	10.9	1.1
$Zn_{0.5}Fe_{2.5}O_4$ (S)	15.6	13.4	12.1 ± 1.2	64.2	1.2	2.2
$Zn_{0.5}Fe_{2.5}O_4$ (L)	26.4	21.1	12.8 ± 2	74.0	5.3	1.6
$Mn_{0.25}Fe_{2.75}O_4$ (S)	-	16	15.7 ± 0.5	80.8	2.1	2.7
$Mn_{0.25}Fe_{2.75}O_4$ (L)	35.1	32.9	18.8 ± 0.5	114.1	7.4	2.0
$Mn_{0.5}Fe_{2.5}O_4$ (S)	15.9	17	-	-	-	-
$Mn_{0.5}Fe_{2.5}O_4$ (L)	27.7	32.7	-	-	-	-
Ferucarbotran[1a]			7.6 ± 4.1	32	-	-
VivoTrax+[2]			21 ± 0.41	108	-	-
VivoTrax[2]			10 ± 0.78	93	-	-

[a] approximately 81% of the ferucarbotran sample had a magnetic diameter of 7.6 ± 4.1 nm, while 19% had a magnetic diameter of 22.1 ± 4.4 nm.

(1) Liu, S.; Chiu-Lam, A.; Rivera-Rodriguez, A.; DeGroff, R.; Savliwala, S.; Sarna, N.; Rinaldi-Ramos, C. M. Long Circulating Tracer Tailored for Magnetic Particle Imaging. *Nanotheranostics* **2021**, *5* (3), 348–361.

(2) Imhoff, E. D.; Melnyk, A.; Rinaldi-Ramos, C. M. Characterization and Evaluation of Commercial Tracers for X-Space Magnetic Particle Imaging. *J. Magn. Magn. Mater.* **2025**, *620*, 172889.
